# Supplementary figures and images for: Nitric oxide mediates neuro-glial interaction that shapes Drosophila circadian behavior
Source: PLoS Genet. 2020 Jun 29;16(6):e1008312. doi: 10.1371/journal.pgen.1008312 (PMC7367490; doi:10.1371/journal.pgen.1008312)

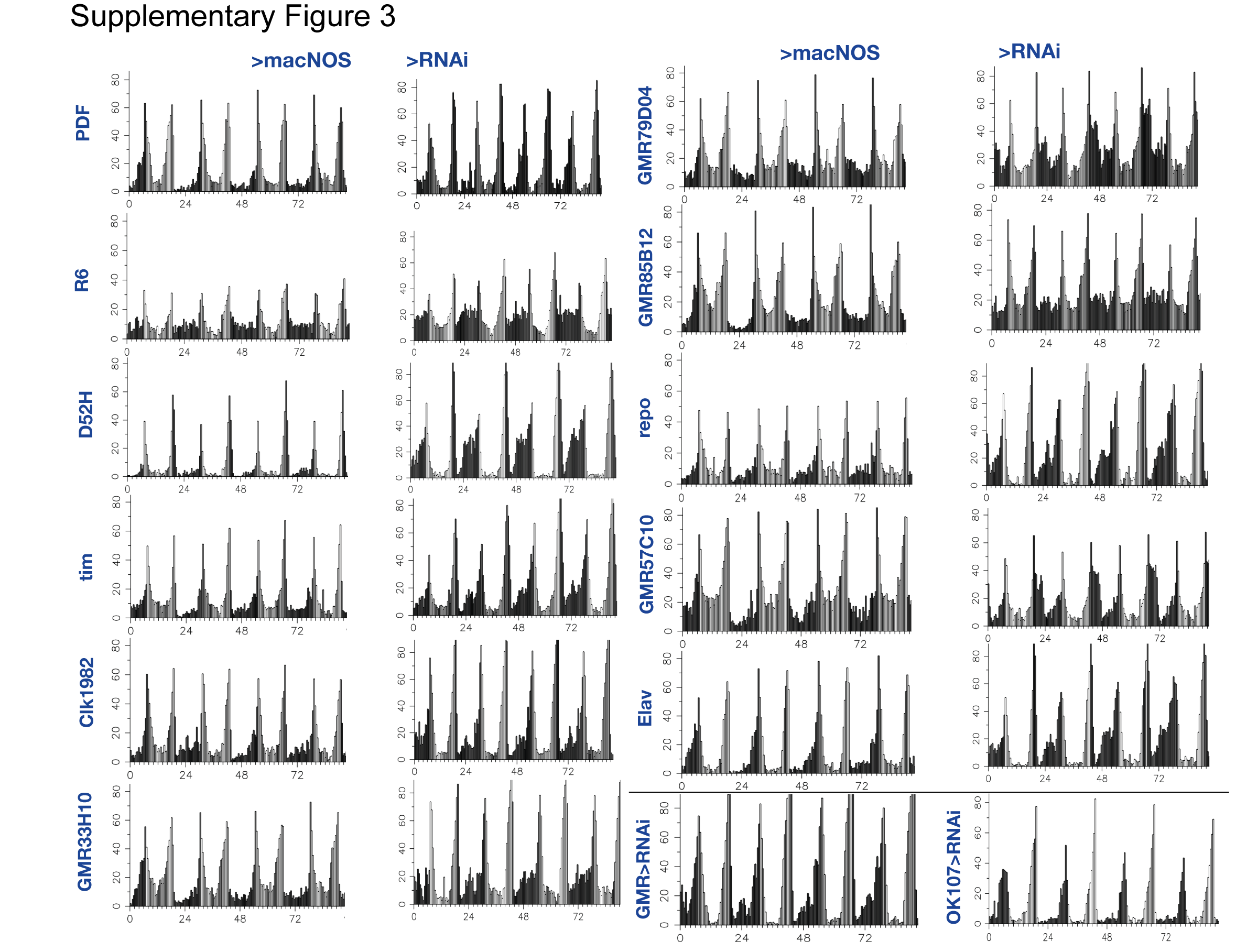

Supplement: S3 Fig — Group average locomotor activity of macNOS or NOS-RNAi27725 expressed with indicated drivers. 4 days of LD are shown. (TIF) [file pgen.1008312.s003.tif]
