# Supplementary material for: Nitric oxide mediates neuro-glial interaction that shapes Drosophila circadian behavior
Source: PLoS Genet. 2020 Jun 29;16(6):e1008312. doi: 10.1371/journal.pgen.1008312 (PMC7367490; doi:10.1371/journal.pgen.1008312)

**Supplementary Table S1. Optic lobe-specific drivers.**

Distribution and intensity of the tested generic optic lobe (OL)-specific drivers, taken from Janelia Fly Light project. Original characterization was based on the GFP expression. Expression of all three OL-specific drivers are enriched in the OL. Expression outside the OL is weak and occasional.


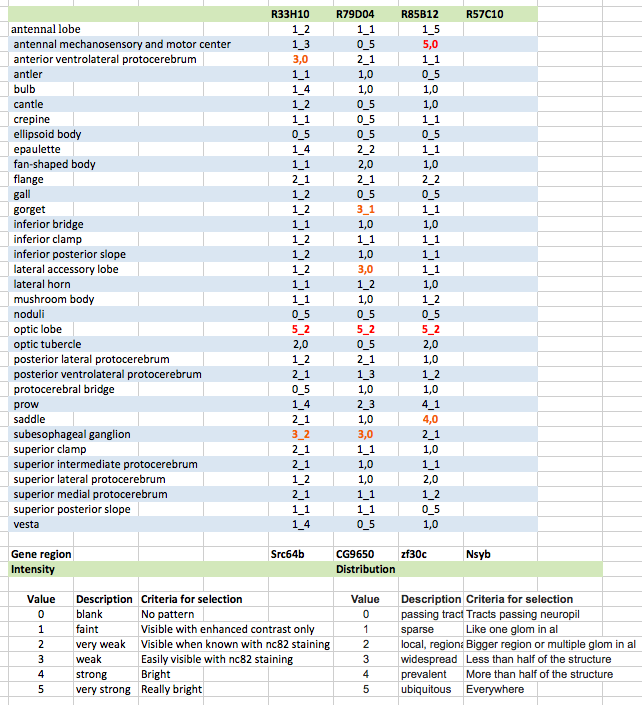

Supplement: S1 Table — Distribution and intensity of the tested generic optic lobe (OL)-specific drivers, taken from Janelia Fly Light project. Original characterization was based on the GFP expression. Expression of all three OL-specific drivers are enriched in the OL. Expression outside the OL is weak and occasional. (DOCX) [file pgen.1008312.s005.docx]
