# Supplementary material for: Nitric oxide mediates neuro-glial interaction that shapes Drosophila circadian behavior
Source: PLoS Genet. 2020 Jun 29;16(6):e1008312. doi: 10.1371/journal.pgen.1008312 (PMC7367490; doi:10.1371/journal.pgen.1008312)
Supplement: Attachment — Submitted filename: Response_NOS_070220.pdf [file pgen.1008312.s006.pdf]

Response to Reviewers

Reviewer #1: Comments to Kozlov and Nagoshi.
The manuscript by Kozlov and Nagoshi describes the involvement of the signaling molecule nitric oxide (NO) in the fly brain, and particularly, its relevance to different outputs of the small LNvs. The authors characterize rhythmic locomotor activity and structural plasticity in two deletion mutants and propose that the structural aberrations trigger the behavioral phenotypes without affecting the molecular clock. Such effects appeared not to be cell-autonomous, and the authors set out to look for the source of the NO signal through Gal4 directed overexpression of a constitutive version of the NO synthase or RNAi-mediated downregulation. As the authors recognize in the text, NO plays a role within the mammalian SCN, and it would be interesting to investigate its relevance to the Drosophila circadian network further. However, despite the inherent difficulty of analyzing the impact of a diffusible signal in vivo, there are several interpretations that need to be toned down and further confirmed.

We thank the Reviewer for constructive comments. Following the suggestions made by the Reviewer, we performed additional experiments, removed inconclusive data and eliminated overstatement in the revised manuscript.

Specific comments.
In Figure 1, panels B and C. Statistical comparisons of 3 genotypes -even if carried out pairwise- cannot be performed by a Student´s t test. Please revise the statistical analysis throughout the manuscript.

In fact, the data were analyzed properly using ANOVA with multiple-comparisons test but the legend was wrongly written by mistake. Thank you for spotting this. Additionally, we have revised statistical tests throughout the manuscript and corrected where necessary.

Figure 3. Panel A. Both mutants show aberrant projections; however, despite both are loss of function alleles the phenotypes vary a great deal: in addition to some miss-routing of specific terminals and the presence of secondary or tertiary neurites, the integrity of the membrane itself appears damaged, particularly so in the representative examples shown for NOS(delta)all. Is there any explanation to such a difference when both mutations apparently give rise to a similar reduction (Fig. 1C)? Also, have the authors quantitate PDF levels in those samples? That would be an important control to establish the link between the morphology of the terminals and rhythmic behavior.
Likewise, the authors mention that loss of NO signaling during development could affect the correct wiring and thus be responsible for the defective behavior, but this possibility was not tested (Given the relevance of this claim to the take home message perhaps it should be).

As suggested, we repeated the experiments and measured both axonal morphology and PDF levels. We confirmed the significant increase in overall length of axon terminals, severely disrupted forms and some “blobs”, and also increase in PDF levels in NOS mutants (new Fig 3A-C). After inspecting many images, we concluded that morphological phenotypes are very similar between NOS (delta)all and NOS (delta)ter mutants. As stated in the text (line 172-175), we failed to detect axonal structural rhythms even in the control group. But apparent rhythms of axonal structure are notoriously sensitive to any genetic manipulation. Since PDF rhythms (which we believe to be more accurate indicator of s-LNv output) are

impaired in mutants, we think it is fair to conclude that overall morphology and rhythmic output of the s-LNvs are severely impaired in NOS mutants.

To distinguish the role of NO signaling during the development and adulthood in the s-LNv structure and function, we also quantified PDF levels in the s-LNvs in adult-restricted NOS knockdown in glial cells. Although this manipulation lead to behavioral arrhythmia comparable to those in NOS mutants (Table 1), PDF rhythms were not affected (Fig. 6A). This new piece of data support our conclusion that structural (and likely wiring) abnormality is caused by the loss of NO signaling during development.

Another interesting observation is described in Fig 2. In terms of the behavioral phenotypes, aside from the startle effect Panel B shows that mutants mostly lack the anticipatory activity to lights off (the evening peak), as opposed to lights on. How can this be reconciled with a defective output of the neurons controlling the M peak? Please explain.

To re-evaluate LD behavior of NOS mutants, we analyzed circadian behavior of another NOS null mutant, NOS(delta)15 and NOS(delta)15/Df. They show a high proportion of arrhythmicity in DD and impaired morning anticipation. However, evening peak was not consistently affected across mutants in different genetic backgrounds (Fig. 2). These data collectively indicate that loss of NOS activity impairs the function of M-cells,

The authors test whether the transcription factor UNF would be involved in the defective NO response that triggers the structural defects; however chronic expression of UNF RNAi in the LNvs gives rise to highly arborized terminals, this phenotype does not resemble at all the ones described in the mutant. How can this be reconciled? Additional, more specific, comments are included below.

We agree with your concern and, since we could not pinpoint the mechanistic link between NO and UNF/E75, we removed the data (previously was in Fig S2) from the revised manuscript. However, we still think that NO regulating UNF/E75 is a valid hypothesis worth entertaining, we mention it in Discussion (line 361-364). Please also see the "Additional Data" below in the response to Reviewer 2. These are our preliminary data addressing this possibility.

If PDF neurons do not express NOS (according to Table I), how relevant is that macnos expression triggers a clear phenotype in the molecular clock (Fig 4D)? Could this be an indirect effect of affecting PDF levels? How do the arborizations look under these conditions?

macNOS expression in the s-LNvs only mildly affects behavioral rhythms. However, when expressed in several other cell types, rhythmicity was more severely affected (Table 1). Overall, this set of experiments shows that forced production of NO is detrimental to behavioral rhythms. We did PER and PDF double staining to evaluate the molecular rhythms in the s-LNvs, LNds and DN1s in pdf>macNOS flies. As shown in Fig. 5A and B, there was a phase-shift in PER rhythms in the s-LNvs but not changes were detected in other clock neurons. Therefore, it is unlikely that PDF transmission is affected in this condition. PDF levels were not apparently affected, though data are not shown as we think this information

is not essential. We did not quantify the axonal arborization in this experiment for the same reason.

To identify potential sites of NO production macNos OX and Nos KD was attempted in different groups of cells. Interestingly, they found that both treatments trigger a behavioral phenotype when expressed under a panglial promoter (Repo), a pancircadian promoter (tim), as well as a panneuronal one (GMR57C10). How can this be explained? In addition, immunohistochemistry should be performed –ideally in chronic and adult- specific fashion) to corroborate the link between the behavioral phenotypes and the complexity of the sLNv projections.

To address these important points, we did two sets of new experiments. (1) Adult-restricted NOS RNAi with the pan-glial Repo-GAL4 driver and anti-PDF staining at two timepoints to evaluate PDF levels and s-LNv projection patterns. We found no apparent changes in PDF levels, rhythms and projection are (Fig. 6A). (2) NOS RNAi with GAL4 drivers targeting different glial subtypes and behavioral assays. Strikingly, we found that only the driver that targets subperineurial glia caused behavioral arrhythmia (Table 2, Fig 6B and C). The perineurial glia (PG) are known contain circadian clocks.

These new pieces of data clarified that NO produced in glia in adulthood does not affect rhythmic s-LNv output (PDF and projection complexity), and thus likely to impact on circadian locomotor output circuit. Furthermore, the fact that PG have circadian clocks explains why NOS RNAi with Tim-GAL4, Repo-GAL4 and PG-GAL4 all similarly reduce locomotor rhythmicity. NOS RNAi with a pan-neuronal driver in adulthood only affects period length but not rhythm strength (Table 1). These results altogether suggest a presence of neuronal cells that affect frequency of locomotor output, and indicate PG as a major source of NO that is required for the generation of locomotor rhythms.

There is room for improvement in figure legends (all, in the main text as well as the supplementary one) and in the figures themselves. Figure legends do not provide critical information to assess the experiments; labeling within the figures is also scarce.

Agreed and thoroughly revised figure legends and figures in the revised manuscript.

Additional comments.

Fig. 1 panel C. The y axis is such that the curves describing DARM staining in both mutants is difficult to see. Could that initial segment be expanded?
Legend to Supp Table I. Page 24, line 661, please revise ….¨in compare with¨

The DAR4-M signal in the mutants are extremely low, barely above the background. So we think it is not important to modify Fig.1C. We also corrected the grammatical errors in the Table S1 legend.

Figure 2. In panel A, mutant activity profiles are compared to w1118 control, while in panel B are compared to CS. What is the genetic background of these mutants? Please clarify or include the proper genetic controls. There are several phenotypes associated to the knock-down NOS alleles, a reduced startle effect to lights on/off, a decreased evening anticipation, and even reduced overall activity. Shouldn´t some of the phenotypes (i.e. quantitation) be included in this main figure?
The grey shading is missing in the first day, before lights on.

The genetic backgrounds of these mutants are not known, but two CRISPR mutants were generate from the same background. Therefore, we added another NOS null mutant (delta 15) generated from a different background in homozygous and hemizygous to our analysis. Altogether, we found that the most consistent phenotype across all mutants is the low rhythmicity in DD and reduced morning anticipation. We show behavioral data summary of heterozygotes for all mutants in Table 1, but we did not show the activity profiles in Fig.2 to avoid overclouding the figure.

Thank you for commenting on LD phenotypes of NOS knockdown alleles (Fig. S3). In the revised manuscript, we briefly describe the phenotype in LD behavior caused by NOS knockdown with different driver (line 271-274). However, since the current study focuses on DD behavior, we decided to keep the data in the supplemental figure. Thank you also for spotting the missing grey shade (corrected).

Fig 2 legend. N is usually employed to refer to the number of experiments performed, and n to the number of animals tested. I would suggest changing to lowercase, and describe in the legend how many experiments were carried out in each case.

Thank you for pointing out this mistake. We corrected it, and wherever appropriate indicated sample numbers and repeats of experiments in the legend, figure, or in the method.

Supplementary Fig 2. The figure includes ¨representative images¨ at ZT2, presumably from 3 independent animals, which should be detailed in the figure legend. Also, is it possible that the labels are mistaken? The ¨control¨-labeled ones clearly do not look normal projections at ZT2. The authors could do a better job at describing the terminals (complexity/degree of arborization using Sholl analysis or the Image J plug in; the length of principal neurites, etc).

As stated above, we realized that this piece of data is not relevant for the study of NOS and thus removed the Fig S2.

Figure 4, panel C. The image shows a single LNv stained for DAR4M above background. Was this always the case (a single small LNv/hemisphere)? That would indeed be interesting, as not too many differences within the cluster have been described. At what timepoint was the staining performed? Panel D, left. Please include labels indicating the times each image refers to.

We generally found more than one cell labeled with DAR4-M in the s-LNs cluster but it was difficult to obtain clear images since DAR4-M staining is technically very challenging. The picture shown in the Fig. 4 was taken at ZT0 (indicated in the legend in the revised manuscript), but we detected the staining at all timepoints tested (ZT0, 6, 12 and 18).

Reviewer #2: This study is aimed at understanding the role for Nitric Oxide Synthase (NOS) in the control of circadian behavioral rhythms in Drosophila. Partial loss of rhythmicity is observed with both mutants tested, and with RNAi driven by different GAL4 drivers. The authors propose that glia is a likely important source of NO. This would establish a novel role for glia in the regulation of circadian behavior. Interestingly also, dorsal projections of the sLNvs, the main circadian pacemaker neurons, are defective in NOS mutants, and this might explain the partial loss of rhythmicity. Overall, this study is innovative, but the authors need to better support their claims. For example, it would be important to make sure that the phenotypes indeed map to the nos gene. Also, the model that NO would work through E75/UNF is very exciting, but direct evidence is lacking.

Thank you for your constructive comments and suggestions.

Major comments:

1) I am puzzled by the fact that the complete deletion of the nos gene has a significantly weaker phenotype than the partial deletion, both in LD and DD. Why is it so? Is the partial deletion predicted to eliminate all NOS isoforms? If so, the two alleles should behave similarly. Could genetic background be an issue? I presume both mutants were generated from the same genetic background, so the absence of complementation in nos-ter/nos-all flies could be the result of a secondary mutation. Unfortunately, rescue with the GAL4/UAS system is not an option since overexpression causes arrhythmicity as well. Thus, I would suggest to validate the observations with backcrossing (unless this was actually done) and by crossing the mutants to deficiencies to map the phenotype to the chromosomal region of NOS.

We agreed with your concern and analyzed behavior of two additional NOS mutant alleles, NOS(delta)15 and NOS(delta)15/Df, to the behavioral analysis. We found that all mutants alleles consistently show low rhythmicity in DD and reduced morning anticipation (Fig. 2, Table 1). Therefore, we are confident that the abnormal circadian behavior is due to the loss of NOS gene.

2) Is the morning peak of activity also eliminated or reduced with RNAi?

Thank you for commenting on LD phenotypes of NOS knockdown alleles. The short answer is that there are differences across different alleles, although they all show bimodal activity patterns (Fig. S3). In the revised manuscript, we briefly describe the phenotype in LD behavior caused by NOS knockdown with different driver (line 271-274).

3) The complete deletion results in 50% of flies being arrhythmic, while the rhythmic flies seem quite normal based on power. Are the average activity plots an average of both rhythmic an arrythmic flies? What happens to the morning anticipation if the rhythmic and arrythmic flies are separated? Is it completely missing in arrhythmic flies, but present in rhythmic flies? Does this correlate with the severity of the defect in synaptic projections? It would be worth staining brains of rhythmic and arrhythmic flies to see if there is a correlation with the severity of the sLNv projection defects.

These are excellent suggestions. We now show the activity plots of rhythmic and arrhythmic flies separately (Fig. 2). Morning anticipation activities tend to be more disrupted in arrhythmic group but it was not all-or-none. We re-analyzed the morphology of s-LNv projections and PDF levels; there was no clear heterogeneity among the brains of the same genotype (Fig. 3A-C).

4) There is clear defect in the sLNv projections, but I have a hard time seeing the rhythms in projection complexity in the control flies (fig3). Quantifications would be helpful here. Also, length was measured, but is thickness also affected. Number of branches?

As suggested, we repeated the experiments and measured both axonal morphology and PDF levels. We confirmed the significant increase in overall length of axon terminals, severely disrupted forms and some "blobs", and also increase in PDF levels in NOS mutants (revised Fig 3A and B). However, as stated in the text (line 172-175), we failed to detect axonal structural rhythms even in the control group. Rhythms of axonal structure are notoriously sensitive to any genetic manipulation. Since PDF rhythms (which we believe to be more accurate indicator of the s-LNv output) are impaired in mutants, we conclude that overall morphology and rhythmic output of the s-LNvs are severely impaired in NOS mutants.

5) I presume that on figure3A pdf-gal4 was used, as in figure S2. This should be indicated. But what are the more or less circular structures seen in the nos mutants, particularly in nos-ter? Some are quite big. Again, it seems overall severity of the phenotype is more severe in nos-ter than nos-all. This correlates with behavior phenotypes, but not with the severity of the genetic lesion.

We indicate the genotypes in the legends in the revised Fig, 3A (gal1118>mCD8). We also wonder what these "blobs" are but cannot find any explanation.

> Again, it seems overall severity of the phenotype is more severe in nos-ter than nos-all.

We realized that the pictures we have picked to show in the original manuscript were not very representative. After inspecting many samples, we found that s-LNvs in NOS(delta)ter and NOS(delta)all mutants are both equally severely affected. The quantification supports this impression (Fig. 3B and C). Revised Fig.3A shows more representative images.

6) The model that NOS would work through UNF/E75 is very interesting. Could it be tested

through genetic epistasis, or with double heterozygotes? Or is there a reporter for UNF/E75 that could be used in sLNvs?

We agree that none of the experiments directly tested the hypothesis that NO inhibits UNF/E75 dimerization thereby controlling PER rhythms in the s-LNvs. Genetic experiments to test this hypothesis was unfortunately possible from a number of reasons (RNAi, overexpression of UNF and E75 both cause strong behavioral phenotype, NOS, E75, UNF genes are haplo-sufficient, since NO is diffusible, manipulating E75 and UNF in the s-LNvs while NOS has to be ubiquitously manipulated, etc).

To more directly examine the effects of acute increase in NO on molecular clocks, we took advantage of *ex vivo* brain culture system that allows for pharmacological manipulations of the medium and timelapse imaging of fluorescent reporters (Sabado et al. 2017 Sci Rep). We used the NO donor diethylenetriamine (DETA) to increase NO production and monitored its effect on clock gene transcription using the fluorescent reporter 3x69-VNP, which expresses a short-lived variant of VENUS under the control of clock response sequence (CRS) of *per*. UNF and that E75 bind the CRS and enhance CLK/CYC-mediated transcription (Jaumouillé 2015 et al, Curr Biol). The addition of DETA decreased 3x69-VNP levels in the s-LNvs over the course of several hours (please see the Additional Data below). This result is consistent with the notion that high levels of NO disrupt UNF/E75 dimerization. However, since NO has numerous biological targets, it is equally possible that NO inhibits CLK/CYC-mediated transcription independently of UNF/E75. Therefore, we decided to not to display this data but added a brief discussion on this possibility in Discussion (line 361-364).

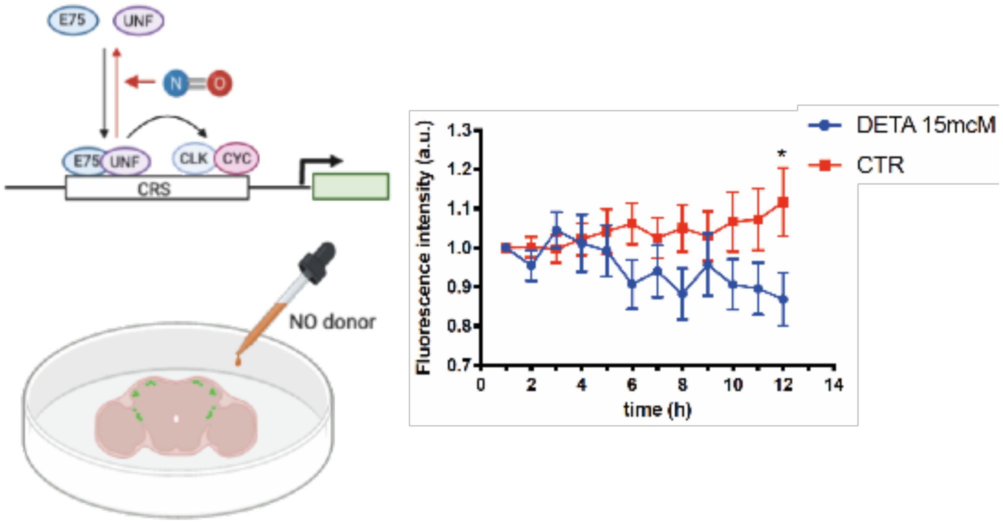

Additional Data.
Adult fly brain explant culture and live-imaging of 3x69-VNP reporter were performed as described previously (Sabado et al. 2017. Sci Rep). Newly hatched flies expressing the 3x69-VNP reporter were entrained to LD cycles for 3 days and the brains were dissected at ZT12 and cultured. The culture was immediately transferred onto the stage of a Leica TCS SP5 tandem scanner confocal microscope for time-lapse imaging. 1 h after the start of the live imaging, NOS donor DETA (15μM) or vehicle (H_2O) was added to the medium. Addition of DETA decreased the VNP expression levels. (*$p<0.05$ with two-way ANOVA.)

7) That the UNF-RNAi and nos mutant projections phenotypes are similar is not obvious to me. The projection lengths in UNF RNAi appear shorter than control, while in nos mutants they are longer. Moreover, it seems the projections are thinner and less structured in nos mutant than UNF. Perhaps this is reflecting the quality of the staining in the two experiments, but again, some form of quantification would be important.

We agreed with your concern. In addition, since we could not pinpoint the mechanistic link between NO and UNF/E75 as explained above, we removed the data (previously was in Fig S2) from the revised manuscript.

8) Lines 221-223 are not very clear, but after reading lines 331-332 in the discussion, I see what the authors meant. However, if UNF/E75 increase per transcription, and if overexpression of NOS increases UNF/E75 dimerization, is this not supposed to advance PER accumulation and shorten period, rather than delaying PER accumulation? Increased per transcription leads to short period (Kadener 2008), and the senior author's group showed that losses of UNF or E75 in adult lengthen period. Therefore, the NOS overexpression phenotype does not seem to fit well with the idea that it works though UNF/E75. As mentioned above, it would be important to gather additional support for this idea

Please see the answer to the point (6).

9) I would suggest adding the timGAL4/repoGAL80 combination to the RNAi and overexpression studies.

Unfortunately, this cross was semi-lethal.
However, our new finding that the perineurial glia (PG) are the source of NO important for circadian behavior (Table 2, Fig. 6B-D) is consistent with the result of tim>NOS RNAi, as PG contain molecular clocks.

10) Why does pan-neuronal RNAi cause a long period phenotype, but not the complete knock-out? Could this be an off-target effect? A possible way to check this is to perform the RNAi experiment in a nos null background. There should be no period lengthening there. A second RNAi line would be an alternative.

It is a good point, but we cannot explain why. It is possible that mutants affect development and wiring of many different neurons, which might have “compensated” the long-period phenotype caused by the loss of NOS in neurons. Addressing this possibility is out of the scope of this paper but is an interesting issue for future studies.

Minor:

1) Line 233-234: Is molecular cycle phase in the sLNvs really misaligned with the rest of the circadian neural network? This would be interesting to know

Yes. We now include the data of PER rhythms in the LNds and DN1s (Fig. 5B).

2) Nos mRNA rhythms should be tested under DD too.

Since we have no data showing that rhythms of NO production are important for its function, we think that it is unnecessary to perform this experiment. Especially, since Sehgal lab has shown that molecular clocks in the perineurial glia are unimportant for locomotor rhythms, even if NO production from perineurial glia is clock-controlled, the rhythms are not relevant for the function. In the revised manuscript, we state that dNOS1 mRNA rhythms were observed “at least in LD” to avoid overstatement.

3) Is there a reference to support the idea that the R57C10 driver is stronger than elaV (line 244-45)?

We added a reference (Sims et al. 2019 eLife) that describes this issue.

4) In the adult specific knock-out, the GMR84B12 control was very arrhythmic, so the level of arrhythmicity in the RNAi cross is not meaningful. It might be best to remove these flies from the table.

Agreed and removed GMR84B12 from Table 1.

5) Figure 2B, legend. A graph is shown, not an histogram as indicated in the legend.

We corrected the figure description.

…………………..

Reviewer #3: Kozlov and Nagoshi investigated role of nitric oxide synthase (NOS) in regulation of circadian behavior in Drosophila. First they found mutants lacking NOS have defect in circadian locomotion. The same mutant also showed developmental defect of the key pacemaker neurons. In cultured brains, glial cells exhibited strong DAR4-M signals, dye sensor that become fluorescent after reacting to NO. Together with glia specific knockdown of NOS in adult flies, they concluded that NOS is active in adult brains and is an essential mediator of neuro-glial interaction in circadian system. These findings can be of general interests, but need to be validated with additional control experiments.

Thank you for your suggestions. We believe that, by performing additional experiments suggested by the Reviewer, we could significantly improve the manuscript.

Major
1) In which glial cell type NOS is expressed? Resolution of genetic dissection is insufficient

to gain mechanistic insights about interaction between specific type of glia and neurons. Kremer et al., GLIA 2017 (https://doi.org/10.1002/glia.23115) reported many driver lines for distinct types of glia. Authors should address which type of glia express NOS by RNA-Seq using these driver lines or FISH experiments. Cite papers if there is any published RNA-seq or quantitative PCR data for supporting NOS expression in glia. Davis et al., reported that there is no NOS expression in glia in the optic lobe (http://dx.doi.org/10.1101/385476).

To address these important points, we conducted NOS RNAi with GAL4 drivers targeting different glial subtypes and behavioral assays. Strikingly, we found that only the driver that targets subperineurial glia caused behavioral arrhythmia (Table 2, Fig 6B and C).

In addition, we performed DAR4-M staining on the brains of the flies expressing GFP with Repo-GAL4. The results clearly showed co-localization of DAR4-M and Repo-GAL4-expressing glia (Fig. 4B).

2) Following (1), validate that NOS proteins express in defined glial cell type by immunohistochemistry, and NOS immunoreactity can be reduced by NOS RNAi knockdown. Antibodies may not distinguish splicing isoform of NOS, but it depends on which splicing isoforms are expressed. Also it is important to examine where in the glial cells NOS localize.

Please see the answer to point (1).

3) Interpretation of DAR4-M signals requires additional control for distribution of dye itself. Given the architecture of insect brains, DAR4-M needs to pass through glia to penetrate to neuropils. Thus, seemingly glia specific signals of DAR4-M could be because DAR4-M was stuck in glia and did not penetrate inside neuropils. DAR4-M fluorescent signals need to be normalized by local concentration of DAR4-M. After loading DAR4-M, fix brains and exposing them to chemical donor of NO such as NOC-7 to examine loading of DAR4-M.

We did realize that DAR4-M staining is technically very challenging. We clearly detected DAR4-M signal in surface glia, which turned out to be the most important source of NO for circadian locomotor behavior. Therfore, we believe that our method was sufficiently specific to estimate the site of NO production.

4) Page 8, line 200: “The signal was particularly high within and around the central complex and in the optic lobe” Provide more detail anatomical description and images.

As shown in the Fig. 4, DAR4-M signal was found throughout the brain. We don’t think describing more anatomical details will add more information. As stated in the Results (line 196-207), the image is largely in agreement with the previous immunohistochemical studies of NADPH diaphprase activity and sGC/cGMP. Additionally, we show the picture of DAR4-M/Repo>GFP double labeling in the revised manuscript (Fig. 4B)

5) Regulski and Tully reported that NOS in Drosophila is calcium/calmodulin dependent

(https://doi.org/10.1073/pnas.92.20.9072). Thus it is important to consider how NOS could be activated in glia. Is calcium dynamics in cultured brains comparable to that of physiological state? Is it possible that DAR4-M fluorescent signals in glia could be due to NO released from neurons?

As stated above, our DAR4-M staining and RNAi data are congruent and show that NOS within the perineurial glia controls locomotor rhythms. Calcium dynamics could be different between cultured brains and *in vivo*. However, since brain culture was used only to verify the loss of function in NOS deletion mutants, we do not think it is an essential point to discuss in this study.

6) If glial NOS is primary source of NO, NOS-RNAi with repo-GAL4 but not 57C10-GAL4 should reduce DAR4-M fluorescent signals in glia.

Our statement may have been confusing in the original manuscript. The point is that the major source of NO that regulates circadian behavior is glia (specifically the perineurial glia), but we never meant to suggest that most NO is produced in glial cells. We restated this conclusion more clearly in the revised manuscript.
