# Supplementary material for: Nitric oxide mediates neuro-glial interaction that shapes Drosophila circadian behavior
Source: PLoS Genet. 2020 Jun 29;16(6):e1008312. doi: 10.1371/journal.pgen.1008312 (PMC7367490; doi:10.1371/journal.pgen.1008312)
Supplement: Attachment — Submitted filename: Response to Revieweres and Editors.pdf [file pgen.1008312.s007.pdf]

Response to Editors

There are 2 main issues raised by the reviewers to the revised version:

1- Issue of the role of NO during development
Your work shows that KD (knockdown) of NOS restricted to the adult causes arrhythmicity yet does not affect the arborization of PDF neurons. This suggests that the anatomical defects shown in Fig. 3A are caused by KD of NOS during development; yet, this is never demonstrated directly. We understand your reticence to performing additional experiments, but if you were to find that KO of NOS during development caused anatomical defects similar to those shown in Fig. 3A, but did NOT affect behavioral rhythmicity it would mean that changes in PDF arborization do not necessarily affect rhythmicity. This would, in itself, be of interest to the "Drosophila clock community" since much has been made of the importance of the daily changes in arborization of PDF neurons. Should you be willing to perform these experiments we assure you that the normal 1 month time limit could be extended if requested; after all, we are no longer living in normal times....
If you were not willing to perform these experiments then we would ask you to please revise the text and remove any suggestion that you looked at the role of NOS during development. For example, in Lines 164-165 you state: "Since low rhythmicity in DD and poor morning anticipation are indicative of the dysfunction of the s-LNvs, we sought to examine possible effects of NOS loss-of-function on the development of the s-LNvs." However, the experiment described did not limit NOS KD only to development but were done for the entire life of the fly including adulthood. We would also suggest that you remove most of the text where you mention a developmental role for NO as it might lead one to believe that you asked this question for this particular piece of fly behavior.

As suggested, we have rephrased the sentences that describe "developmental" effect of NOS loss-of-function throughout the text. The edited texts are highlighted in yellow.

2- The issue of NOS in surface glia and NO detected in these glia using DAR4M
We agree that you document DAR4M staining in surface glia and that KD of NOS in these glia causes behavioral defects. Yet there is a (formal) possibility that some of the NO detected in surface glia is also produced elsewhere and accumulates in these cells. We agree that this is unlikely and that anyway your findings suggest that surface glia-produced NO plays a role in regulating circadian rhythmicity. However, NO is an unconventional transmitter so we ask that you simply be stricter in your assignment of the source of NO that contributes to the DAR4M you detected in surface glia.

We show that DAR4M-staining detected NO in the surface glia, and NOS knockdown in the surface glia reduces locomotor rhythmicity. Additionally, a previous work by the group of Roland Bainton has demonstrated the expression of NOS in the surface glia using transcriptome analysis (DeSalvo et al. 2014, Front. Neurosci). We strongly argue that these three pieces of evidence indicate

that NOS within the surface glia cells produces NO and contribute to generating circadian locomotor rhythms. This paper is cited in the revised manuscript (This last piece of evidence, already published, was the one asked by Reviewer 3). Nevertheless, we followed your suggestion and toned down this conclusion wherever appropriate. The modified texts with regard to NOS in surface glia are highlighted in blue.

3- Issues raised by the Associate Editor
The Associate Editor has taken the opportunity to make the following suggestions and comments to your manuscript:

a- There are places where the wording used is rather colloquial. We ask that you please revise them. Some examples are listed below (but there may be others; please check your text carefully).
- line 56 "....remains a handy model to study..." "handy" is not a good word choice.
- line 151 "....NOSΔ15 over a deficiency (Df(2L)BSC230)..." We suggest changing "over" for "heterozygous with"
- line 320 "...have an utterly wrong shape..." "utterly" is not a good word choice.

We agree that these colloquial descriptions were inappropriate. We corrected inappropriate word choices and revised confusing sentences throughout the manuscript text. Edited texts are highlighted in green.

b- The records shown in Fig. 2 under "Arrhythmic" do not look particularly arrhythmic to the Associate Editor, especially the 2nd and 3rd record (from the top) show a clear recurring daily pattern. Please either choose better (i.e., more arrhythmic) representative examples, or include the Chi-square periodogram associated with these records.

Thank you for pointing this out. As suggested, in revised Fig.2, we replaced the group-average locomotor activity to representative arrhythmic fly's activity plot. We also include waveforms (for rhythmic flies) and periodograms (for arrhythmic flies).

c- The way the results of ANOVA comparisons are shown makes it quite difficult to read them. This is especially true for Fig. 3B, where there is a jungle of brackets and asterisks. We would suggest that you use different letters to mark the groups that are statistically significantly different.

Agreed and revised the Figure 3B and 3C, removing unnecessary brackets.
