# Supplementary material for: Nitric oxide mediates neuro-glial interaction that shapes Drosophila circadian behavior
Source: PLoS Genet. 2020 Jun 29;16(6):e1008312. doi: 10.1371/journal.pgen.1008312 (PMC7367490; doi:10.1371/journal.pgen.1008312)
Supplement: Attachment — Submitted filename: NOSpaper_Rev3_Cover.pdf [file pgen.1008312.s008.pdf]

UNIVERSITÉ
DE GENÈVE

FACULTÉ DES SCIENCES
Département de génétique
et évolution

Emi Nagoshi, Ph.D.
Associate Professor

Geneva, 2 June 2020

Dear Editors of PLOS Genetics,

We are submitting the third revision of the manuscript entitled “Nitric Oxide Mediates Neuro-Glial Interaction that Shapes *Drosophila* Circadian Behavior” as a research article in PLOS Genetics.

As requested, we improved the quality of images shown in Fig. 4D and Fig. 6A, by changing the background color to white and enhancing the contrast. In Fig. 4D, we show a different picture from the same experiment, as it is more readable. We have also updated the legend of Figure 4D to be consistent with the revised Fig. 4D. The changes made in the revised manuscript text is highlighted using “track changes” in MS word.

We are grateful to the Editors that helped us to strengthen the paper. We hope that you find the revised manuscript is suitable for publication in PLOS Genetics.

Sincerely yours,

Emi Nagoshi, PhD.
Associate Professor, University of Geneva
Emi.Nagoshi@unige.ch

Sciences III - 30 quai Ernest-Ansermet - CH-1211 Genève 4
Tél. +41 (0)22 379 63 46 - Fax +41 (0)22 379 67 95 – Emi.Nagoshi@unige.ch
